# Human activities and densities shape insecticide resistance distribution and dynamics in the virus-vector *Culex pipiens* mosquitoes from Morocco

**DOI:** 10.1186/s13071-024-06164-1

**Published:** 2024-02-19

**Authors:** Soukaina Arich, Najlaa Assaid, Mylène Weill, Fatim-Zohra Tmimi, Hassan Taki, M’hammed Sarih, Pierrick Labbé

**Affiliations:** 1 Institut des Sciences de l’Évolution de Montpellier, UMR 5554, CNRS-UM-IRD- EPHE), Université de Montpellier, Cedex 5 Montpellier, France; 2 Laboratory of Biology and Health, Faculty of Sciences Ben M’Sik, URAC34, Hassan II University of Casablanca, Casablanca, Morocco; 3 Laboratoire des Maladies Vectorielles (LMV), Institut Pasteur du Maroc, Casablanca, Morocco; 4 Institut Universitaire de France, 1 rue Descartes, 75231 Cedex 05 Paris, France

**Keywords:** Resistance dynamics, Anthropization, Insecticide resistance, Mosquitoes, Morocco

## Abstract

**Background:**

Mosquitoes of the *Culex pipiens* complex are widely distributed vectors for several arboviruses affecting humans. Consequently, their populations have long been controlled using insecticides, in response to which different resistance mechanisms have been selected. Moreover, their ecological preferences and broad adaptability allow *C. pipiens* mosquitoes to breed in highly polluted water bodies where they are exposed to many residuals from anthropogenic activities. It has been observed for several mosquito species that anthropization (in particular urbanization and agricultural lands) can lead to increased exposure to insecticides and thus to increased resistance. The main objective of the present study was to investigate whether and how urbanization and/or agricultural lands had a similar impact on *C. pipiens* resistance to insecticides in Morocco.

**Methods:**

Breeding sites were sampled along several transects in four regions around major Moroccan cities, following gradients of decreasing anthropization. The imprint of anthropogenic activities was evaluated around each site as the percentage of areas classified in three categories: urban, agricultural and natural. We then assessed the frequencies of four known resistance alleles in these samples and followed their dynamics in five urban breeding sites over 4 years.

**Results:**

The distribution of resistance alleles revealed a strong impact of anthropization, in both agricultural and urbanized lands, although different between resistance mutations and between Moroccan regions; we did not find any clear trend in the dynamics of these resistance alleles during the survey.

**Conclusions:**

Our study provides further evidence for the role of anthropic activities in the selection and maintenance of mutations selected for resistance to insecticides in mosquitoes. The consequences are worrying as this could decrease vector control capacities and thus result in epizootic and epidemic outbreaks. Consequently, concerted and integrated disease control strategies must be designed that include better management regarding the consequences of our activities.

**Graphical Abstract:**

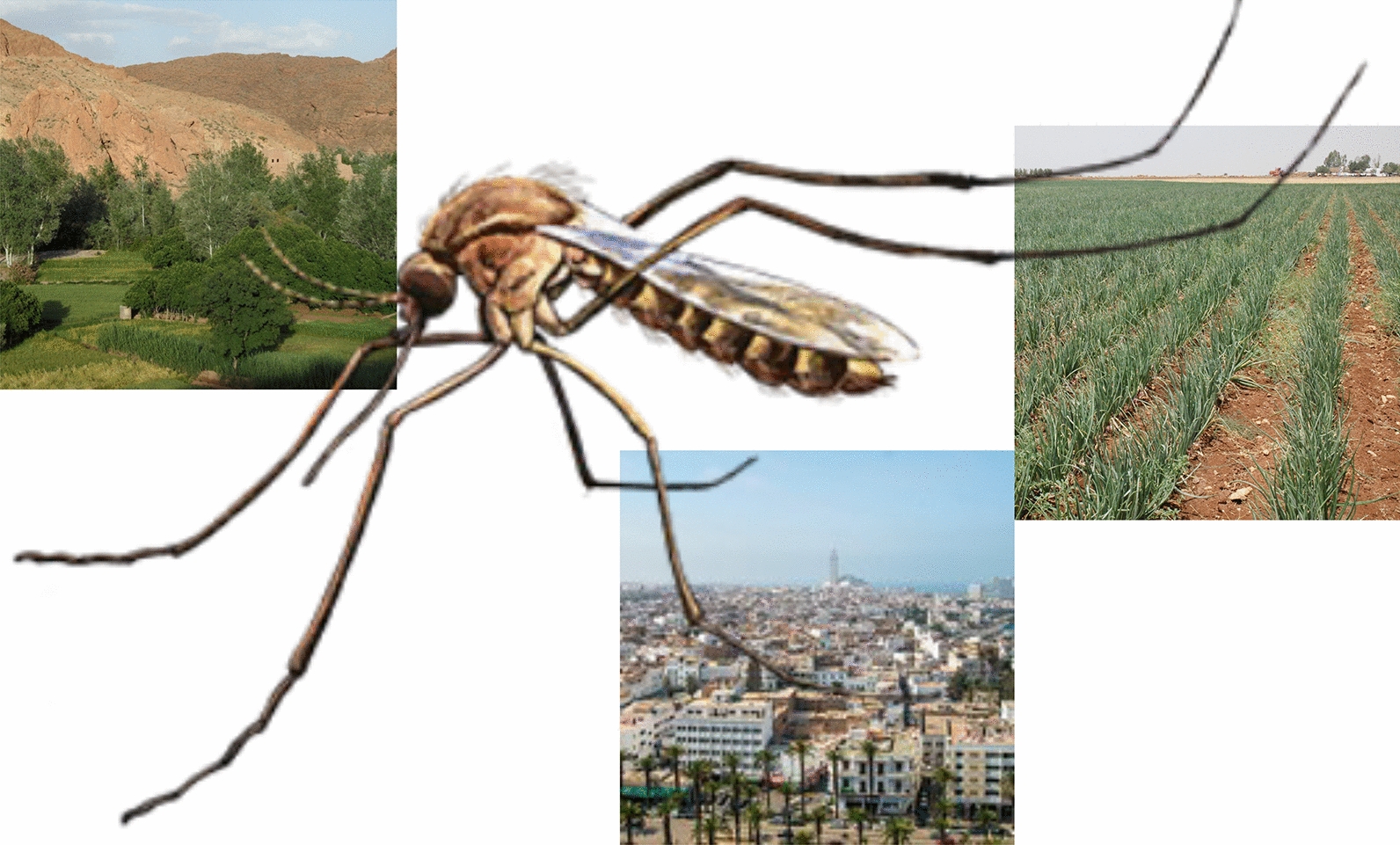

**Supplementary Information:**

The online version contains supplementary material available at 10.1186/s13071-024-06164-1.

## Background

Mosquitoes of the *Culex pipiens* species complex are distributed worldwide, with two main species, *Culex quinquefasciatus* in tropical regions and *C. pipiens* s.s. in temperate regions; they are important vectors of diseases that affect human and animal health [1–4]. In Europe and North Africa (and in North America), *C. pipiens* s.s. is responsible for the transmission of several arboviruses, including West Nile virus (WNV) [5], Rift Valley fever virus (RVFV) [6] and Usutu virus [7], whose reservoirs are birds. For WNV, epizootics in horses and birds, and epidemics in humans, are regularly observed in Europe (2083 confirmed human cases in 2018, including 181 deaths, with a mean of 500 cases per year over the last 5 years [8]) and in Morocco, where several outbreaks of WNV were reported in 1996, 2003, 2010 and as recently as 2021 [9–15].

*Culex pipiens* s.s. is known to be able to breed in a large variety of sites, its highly adaptable larvae being able to thrive in clear ponds as well as highly polluted drainages or sewage farm effluents [1]; this mosquito is particularly abundant in waters charged with high concentrations of organic matter, where it is often one of the very few metazoa able to develop. It is thus no surprise that this mosquito species is often found in water bodies resulting from anthropogenic activities. The ever-increasing imprint of humankind on the environment, or anthropization, includes two main aspects that favor *C. pipiens* s.s.:

i. urbanization [16]: fast-developing cities, particularly in countries where human densities are not controlled, often encounter problems with water effluent pooling and wastewater management, which result in the multiplication of potential breeding sites in close proximity to human hosts [17]. This increases mosquito nuisance caused by their blood-feeding behavior, which can in turn induce a shift in host preferences to humans [18], and also results in more intensive, uncontrolled, domestic use of insecticides [19], both of which can increase disease spread [17].
ii. agriculture [20]: land use modifications for agriculture often include the construction of flooded meadows, ponds, irrigation canals and drainages, which are all favorable environments for the proliferation of mosquitoes. These water bodies also concentrate the residuals from farming activities (animal dejections, pesticides and fertilizers), so that only a few species, including *C. pipiens* s.s., can develop in these conditions, but then in great numbers [21]. Moreover, pesticides used in agriculture and present in these effluents are often similar to those used for mosquito control [20].

Because of both its vector abilities and the nuisance it causes, *C. pipiens* s.s. is the target of insecticide control. In addition, because of its ecological preferences and its broad adaptability, *C. pipiens* s.s. is also exposed to many insecticide residuals. These repeated exposures, both intended and unintended, led to the selection of resistance in this species (e.g. [22]). Resistance to insecticides involves various mechanisms [23–25], the main ones being metabolic resistance and target modification. In *C. pipiens* s.s. (as in most mosquito species), metabolic resistance results from increases in the expression of detoxifying enzymes (glutathione S-transferases, cytochrome P450 monooxygenases, and/or carboxylesterases encoded by the *Ester* locus) that inactivate or sequester insecticides [24–27]. Several insecticide target modifications have also been described that lead to a reduction in susceptibility to insecticides: for example (i) the L1014F “*kdr”* mutation in the voltage-gated sodium channel (encoded by the *vgsc* locus) that causes resistance to pyrethroids (PYR) and DDT (organochlorate or OC family) [28], and (ii) the G119S and F290V mutations in acetylcholinesterase (AChE1, encoded by the *ace-1* locus), allowing resistance to organophosphates (OP) and carbamates (CX) [29, 30]. In addition, some resistance alleles, in particular those implicated in metabolic resistance, allow resistance to several insecticide molecules, sometimes from different families, a cross-resistance that greatly limits the choice of alternative insecticides [27, 31–33].

It has been observed for other mosquito species that both urbanization and agricultural activities can lead to increased exposure to insecticides [34–37], and thus to increased resistance [38, 39], and similar effects were discussed in a few studies for *C. pipiens s.s*. [40–43]. We investigated this in Morocco, a country that has witnessed impressive economic development in recent decades, largely due to the evolution of agriculture in the country (which today accounts for 20% of the gross domestic product [44]). The country development also resulted in rapid and widespread urbanization. Moreover, as *C. pipiens* s.s. is a major disease vector in Morocco, it has been targeted by vector control initiatives. In the 1950s, larval treatments were initiated using DDT (OC), substituted since 1978 by temephos (OP); pyrethroids (PYR, permethrin and deltamethrin) were later introduced as adulticides for epidemic control and are also used for domestic applications [45, 46]. Consequently, resistance alleles to all the insecticides currently or historically used for vector control have been selected [47, 48].

To investigate whether and how anthropization (agricultural lands and urbanization) has had an impact on *C. pipiens* s.s. resistance to insecticides, we sampled several transects of breeding sites in four regions around major Moroccan cities, along gradients of anthropization. As a previous study has shown that these alleles explain most of the resistance observed in Morocco [48], we then assessed the frequency of both target site mutations (*ace-1* G119S and F290V, *vgsc “kdr”* L1014F) and metabolic resistance alleles (*Ester*^*2*^) in 18 samples and followed their dynamics in five urban breeding sites over 4 years. As expected, we found a strong impact of anthropization on the distribution of resistance alleles; we briefly discuss how this should be considered by health authorities to implement effective strategies for vector control.

## Methods

### Study area and mosquito collection

**Fig. 1:**
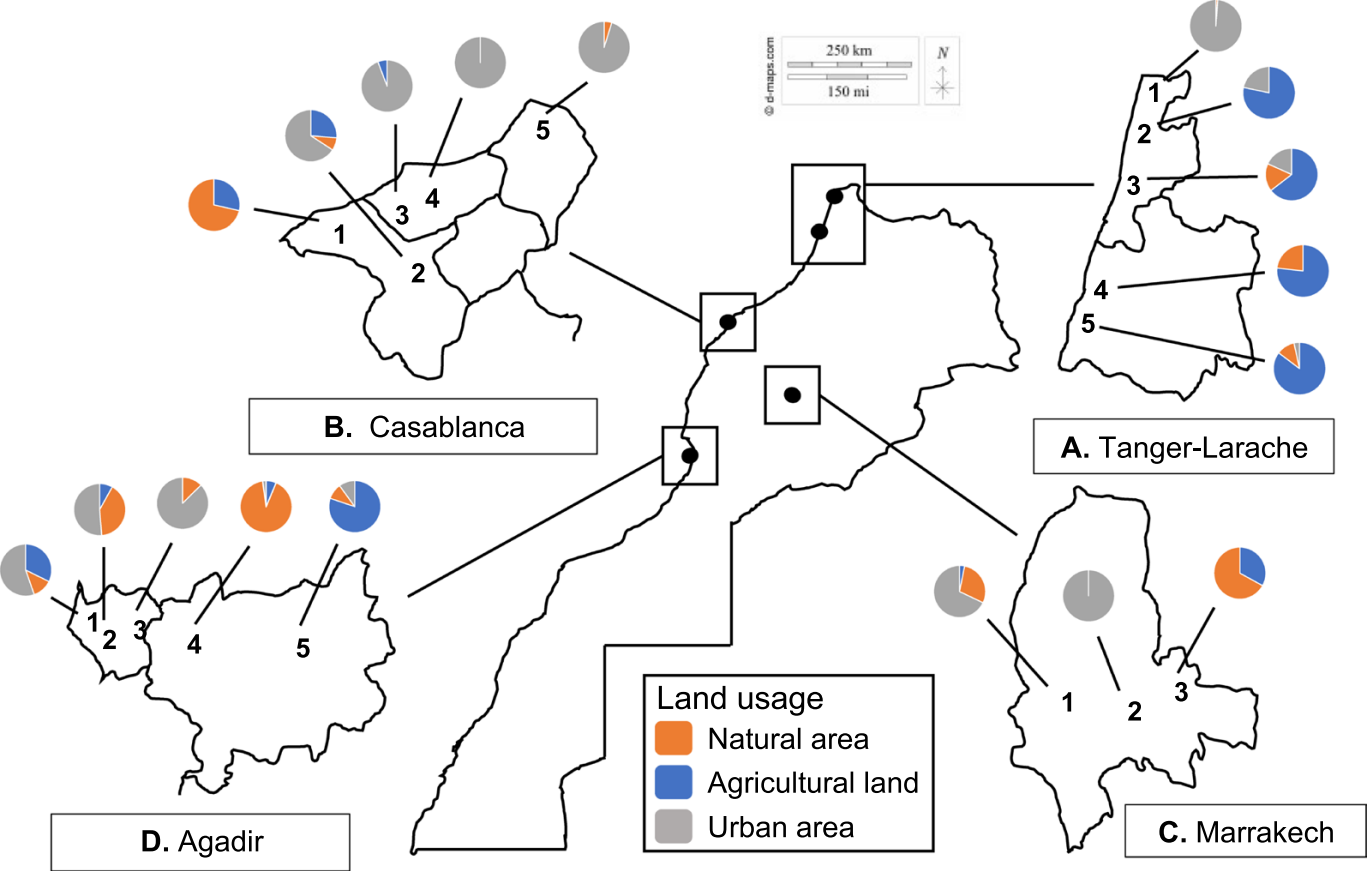
Sampling sites for this study in Morocco. For each region and each sample site, the percentage of each kind of land usage in a 2.5-km-radius area centered on the breeding site is indicated in a pie chart (agricultural land in blue, urban area in gray and natural area in orange)

In four regions across Morocco, transects of three to five populations were sampled during summer 2019, from the urbanized center of main cities to the more rural/natural surroundings, totaling 18 sample sites (Table 1 and Fig. 1). For five of these sample sites, a previous study in 2018 had already described the insecticide resistance alleles present and their frequencies (see Fig. 1) [48]. During the present study, these sites were further sampled over 3 more years (2019, 2020 for Mohammedia only and 2021) to follow their resistance allele dynamics.

**Table 1:** Characteristics of *Culex pipiens* s.s. breeding sites sampled in Morocco

Region		Sampling site	GPS	Types of breeding site	%Natural/agricultural/urban land
					(2.5 km-radius area)
A	Tanger-Larache	***1. Oued Houd***	35°46′44.30N/5°50′50.10W	Raw sewage	1.85/0/98.15
		**2.** Boukhalef	35°44′7.70N/5°53′27.14W	Small pond	0/78.35/21.65
		**3.** Guezenaia	35°41′33.25N/5°55′1.02W	Small pond	17.15/64.57/18.28
		***4. Mezgalef (LS1)***	35°16′48.12N/6°5′13.60W	Wet meadows	23.22/76.78/0
		**5.** Dwar Ain Chouk (LSA)	35°8′55.58N/6° 8′4.91W	Small water basin	11.37/85.17/3.46
B	Casablanca	**1.** Lissasfa	33°29′53.2N/7°43′21.9W	Dirty water pond	71.43/28.57/0
		**2.** Bouskoura	33°27′30.09N/7°37′34.80W	Dirty lake	7.88/26.29/65.83
		**3.** Nassim	33°32′04.2N/7°39′48.1W	Industrial wastewater	0/14.06/85.94
		**4.** Californie	33°32′7.06N/7°36′34.75W	Dirty pond	0/0/100
		***5. Ouled Hmimou (Mohammedia)***	33°40′25.3N/7°26′42.5W	Raw sewage	33.63/39.17/27.2
C	Marrakech	***1. Souihla***	31°68′13.43N/8°17′94.86W	Irrigation canals	67/33/0
		**2.** Saada	31°68′13.43N/8°17′94.86W	Sewer water	29/3.04/67.96
		**3.** Targua	31°38′41.10N/8°3′21.18 W	Flowerpot	0/0/100
D	Agadir	**1.** Aghroud	30°24′45.75N/9°35′56.19 W	Flowerpot	12.6/0/87.4
		**2.** Jorf	30°20′4.53N/9°32′36.23W	Small water basin	12.36/32.28/55.36
		***3. Drarga***	30°22′14.1N/9°29′14.6W	Sewage water	40.68/8.06/51.26
		**4.** Temsia	30°21′43.06N/9°23′43.11 W	Water channel	91.52/6.3/2.18
		**5.** Taroudant	30°30′21.75 N 8°47′26.23 W	Industrial wastewater	9.8/80.2/10

For each sampling site, the region of origin, GPS coordinates, type of breeding site and percentages of each land usage (natural, agriculture and urban) in a 2.5-km-radius area around the sampling site are indicated. The sites sampled in 2018 by Arich et al. [45], and followed over 3 more years, are italicized and bolded

*Culex pipiens* s.s. larvae were collected using the dip-sampling method [49]. Larvae were transported to the insectary for identification using the key for Mediterranean Africa mosquitoes [50]. *Culex pipiens* *s.s. *larvae were identified based on abdominal characters (a single branch of the caudal seta 1-X, 2 to 5 branches of the siphon seta 1a-S and no median spine on the segment VIII scales). Only larvae identified as *C. pipiens* *s.s.* were further used and conserved in 70% alcohol. We collected samples from densely populated larval sites to minimize the possibility of collecting siblings from the same egg rafts.

Using the free *Google Earth pro* software (https://www.google.com/intl/fr/earth/versions/, Google), in which a land cover map of Morocco [51] was projected, circles of 2.5 km radius centered on each breeding site were then drawn. Land usage types within these circles were grouped in three categories: (i) natural areas (categorized as “deciduous broadleaf forest”, “mixed forest”, “shrubland” and “wood land” in the land cover map), (ii) urbanized areas (“built-up land”) and (iii) agricultural areas (“cropland”, “fallow land”, “plantation” and “wheat crop”). Using the software’s *polygon* tool, the percentages of each circle areas devoted to these three land usage categories were then calculated (Table 1 and Fig. 1).

### Molecular analyses

Mosquito DNA was extracted using the cetyltrimethylammonium bromide (CTAB) protocol [52]. For each population, ≈30 mosquito larvae were analyzed by PCR (polymerase chain reaction) tests to characterize their genotypes for the different resistance mutations at three loci: (i) *vgsc* (coding for the voltage-gated Na^+^ channels, the target of PYR insecticides), (ii) *ace-1* (coding for acetylcholinesterase, the target of OP and CX insecticides) and (iii) *Ester* (coding for generalist esterases that cleave insecticides of several families, including PYR and OP). The DNA fragments were separated on 1.5% agarose gel electrophoreses and visualized by ethidium bromide staining under ultraviolet light. A total of 540 larvae were analyzed.

#### Genotyping the *vgsc* locus, "kdr" L1014F mutation

Martinez-Torres et al.’s PASA (PCR Amplification of Specific Allele) diagnostic test was used to genotype the *vgsc* L1014F resistance mutation [53]. Rather than a single combined PCR as described by these authors, we chose to perform two separate PCRs in parallel on each mosquito: (i) one for the susceptible S alleles (L1014, primers Cgd1, Cgd2 and Cgd3) amplifying a common fragment for all individuals and a specific fragment only if the mosquito carries at least one S allele and (ii) one for the resistance R allele (F1014, primers Cgd1, Cgd2 and Cgd4) amplifying a common fragment for all individuals and a specific fragment only if the mosquito carries at least one R allele; combining the two PCR thus allows distinguishing homozygotes from heterozygotes. PCR conditions were 1 min at 94 °C, 2 min at 48 °C and 2 min at 72 °C for 40 cycles.

#### Genotyping the *ace-1* locus, G119S and F290V mutations

Weill et al.’s PCR-RFLP (restriction fragment length polymorphism) diagnostic test was used to genotype the *ace-1* G119S resistance mutation [54]. An exon 3 174-bp fragment encompassing the target site was amplified (primers CpEx3dir and CpEx3rev, PCR conditions: 30 s at 95 °C, 30 s at 52 °C and 1 min at 72 °C, for 30 cycles) and then digested by the restriction enzyme *HaeIII*, according to the manufacturer's instructions (New England Biolabs). The S allele is not digested, whereas the R allele is cleaved into two fragments, which allows distinguishing homozygotes and heterozygotes.

Alout et al.’s PASA diagnostic test was used to genotype the *ace-1* F290V resistance mutation [30]. Depending on the genotype, three fragments can be amplified simultaneously, a 543-bp control fragment (primers CxEx5dir and CxKrev2), a 148-bp fragment specific for S allele’s phenylalanine (primers CxEx5dir and ValRev) and a 435-bp fragment specific of R allele’s valine (primers Valdir and CxKrev2); heterozygotes amplify both specific fragments, homozygotes only one. PCR conditions were 30 s at 94 °C, 30 s at 51 °C and 40 s at 72 °C, for 30 cycles.

#### Detection of the *Ester*^*2*^ Allele at the *Ester* Locus

Berticat et al.’s PCR-RFLP diagnostic test was used to genotype the individuals for the *Ester*^*2*^ resistance allele [55]; this test discriminates mutations specific to the *Ester*^*2*^ allele on both esterases A and B composing this superlocus. A first PCR amplifies an esterase A fragment (primers EstAdir and EstArev; PCR conditions: 30 s at 95 °C for, 30 s at 52 °C and 1 min at 72 °C, for 30 cycles), which is then digested by the restriction enzyme *HaeIII* according to the manufacturer’s instructions (New England Biolabs). A second PCR amplifies an esterase B fragment (primers EstBdir and EstBrev; PCR conditions: 30 s at 95 °C for, 30 s at 52 °C and 1 min at 72 °C, for 30 cycles), which is then digested by the restriction enzyme *HaeIII* according to the manufacturer’s instructions (New England Biolabs). When profiles from both A and B digested fragments correspond strictly to *Ester*^*2*^ profiles, the individual is ruled a resistant homozygote; when both A and B digested fragments display *Ester*^*2*^ profiles, but also other fragment sizes, the individual is ruled a heterozygote; when either A or B digested fragments (or both) do not display the *Ester*^*2*^ profiles, the individual is ruled as susceptible homozygote.

### Statistical analyses

All computations were performed using free *R* software (v.4.1.2, http://www.r-project.org, The R core Team).

For each locus and mutation, a principal component analysis (PCA; function *PCA* in the *FactoMineR* package) was performed to provide an overview of the correlation between the frequency of the resistance mutation (*fR*) and the land usages around the breeding sites (percentages of agricultural, urbanized and natural areas). The 95% confidence intervals for the resistance mutation frequencies were calculated using the function *prop.test* of the *stats* package (*conf.int* values, including a continuity correction).

Generalized linear models were used to test the effects on the resistance frequency (*fR*) of the region of origin of the samples (*Region*) and a variable of land usage (*Land*), either the percentage of anthropization (*i.e*. the sum of agricultural and urban areas) or the percentage of agricultural lands (as the PCA results indicated that these variables were the main drivers of the resistance allele frequencies, depending on the locus considered). The models were of the form *fR* = *Region* + *Land* + *Region:Land* + *ε*, where “:” represents an interaction between the variables and *ε* the error factor following a binomial distribution. GLMs were then simplified as follows: the significance of the different terms was assessed, starting with the highest-order terms (interaction), using likelihood ratio tests (LRTs, function *anova*, χ^2^ tests), and non-significant terms (*p* > 0.05) were removed [56]. When the interaction was found significant (*p* < 0.05), the effect of land usage on *fR* was tested independently for each region: *fR* = *Land* + *ε*. A sequential Bonferroni correction was applied to control for multiple testing [57].

## Results and discussion

Insecticide resistance in mosquitoes is selected as an adaptation to the application of insecticides used to prevent nuisance or vector-borne disease transmission. However, this adaptation can be impacted directly or indirectly by many other alterations of the mosquito ecology associated with human activities and densities. In particular, the development of cities (or urbanization) and agriculture have been shown to have considerable impacts on the spread of resistance in *Anopheles gambiae* [58] and *Aedes aegypti* [20]. Insecticides are indeed widely used in agriculture to control diverse pests, but also select non-target resistant mosquitoes. Domestic use by citizens is not controlled and can play a major role too, with a selective pressure increasing with the population density; similarly, local agencies in charge of disease and mosquito control tend to adjust the insecticide use to the human densities. Finally, the various residuals of human activities (domestic or industrial) may also have cross effects resulting in the selection of alleles originally selected for insecticide resistance (or even select them before the application of insecticides for vector control), and these pollutants also increase in concentration with human density. These non-mutually exclusive sources of selective pressure for alleles allowing insecticide resistance tend to concentrate in the wastewaters inhabited by *C. pipiens* *s.s.*, a mosquito with a proclivity and tolerance for sites with high organic matter concentration. It thus makes it a perfect model to measure the effects of human activities, or anthropization, on the spread of insecticide resistance. In the present study, we performed a temporal and geographical survey of insecticide resistance in *C. pipiens* *s.s. *from Morocco. All data used for the analyses are presented in Additional File 1.

### Agricultural and urbanized lands have a strong effect of insecticide resistance distribution

In summer 2019, we sampled three to five populations along four transects across the country to assess the impact of human activities on the selection of insecticide resistance alleles of three loci.

These transects went from fully urbanized areas to agricultural lands and/or to more natural environments, which represent decreasing densities of human settlements (Fig. 1). For each sampling site, we measured the percentages of land coverage characterized as urban, agricultural or natural in a 2.5-km radius around the sampling point (*%urb*, *%agri* and *%nat*, respectively). The different transects vary substantially in terms of anthropization, the one around Casablanca-Mohammedia being highly urbanized, while the Tanger-Larache transect is more rural (Fig. 1 and Table 1).

Moreover, these transects were designed around five large cities (Casablanca, Agadir, Marrakech, Tanger and Larache), which were previously shown to harbor insecticide-resistant mosquitoes [45]. For each sampling site, we thus measured the frequency of resistant alleles for (i) *vgsc*, with the *kdr”* mutation L1014F providing resistance to PYR insecticides (the main insecticide family used for adult control, including for domestic use), (ii) *ace-1*, with the two mutations, G119S and F290V, providing resistance to OP and CX insecticides (the main family of larvicides used for mosquito control), and (iii) *Ester*, whose resistance allele *Ester*^*2*^ has been found to have spread worldwide [56] and which provides resistance to several insecticide families, including OP and CX.

**Fig. 2:**
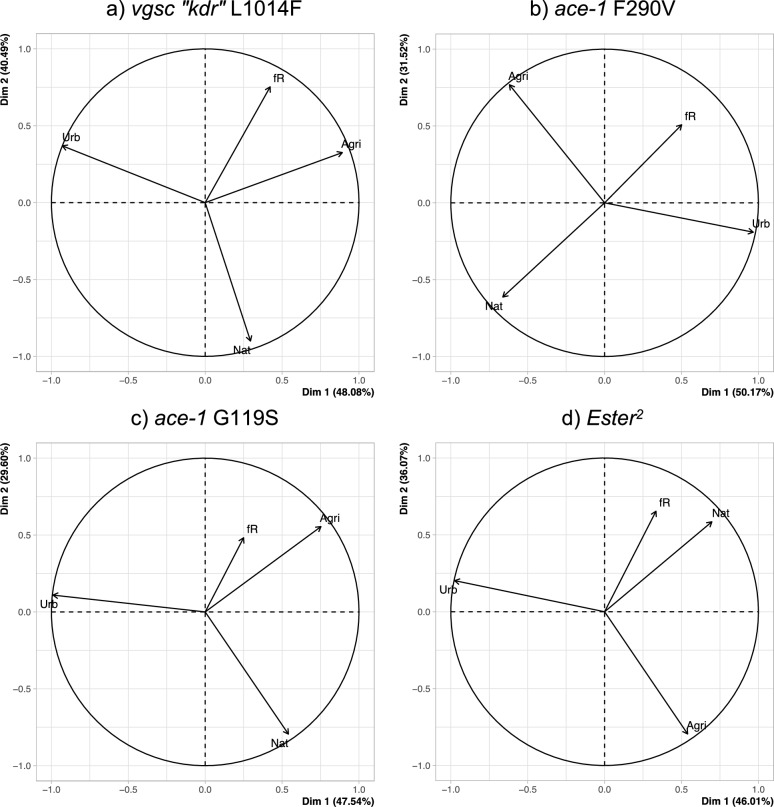
Principal component analysis (PCA) for each resistance mutation. For each resistance mutation **a** vgsc “*kdr”* L1014F, **b** *ace-1 F290V*, **c** *ace-1 G119S* and **d**) *Ester*^*2*^), the variables’ projections on the two first axes of the PCA are indicated by the arrows (for each axis, the explained variance percentage is indicated in brackets). The variables are the resistance allele frequency (fR) and the percentages of natural (Nat), urban (Urb) and agricultural (Agri) areas in a 2.5-km radius area around each sampling site

We then assessed the potential correlations between the resistance mutation frequencies and the land usage variables. As the land usage variables (*%urb*, *%agri* and *%nat*) are not independent (they are complementary percentages of the total area surface around the sampling site, *i.e*. *%urb* + *%agri* + *%nat* = 100%), we first performed principal component analyses (PCAs) for each resistance mutation independently, using the 18 sites sampled in Morocco (Fig. 2) to assess the main tendencies, before properly testing them with GLMs. The first two PCA axes capture most of the variance (about 50% for the first and 30 to 40% for the second axis). Two patterns emerge: (i) for the *vgsc kdr* L1014F and the *ace-1* G119S mutations, the percentage of agricultural land (*%agri*) is the most correlated with the resistance mutation frequency, the two other variables appearing more orthogonal (Fig. 2a, c); for the *Ester*^*2*^ and the *ace-1* F290V mutations, it is the percentage of natural land (*%nat*) that is the most correlated, respectively negatively and positively, with the resistance mutation frequency (Fig. 2b, d).

**Fig. 3:**
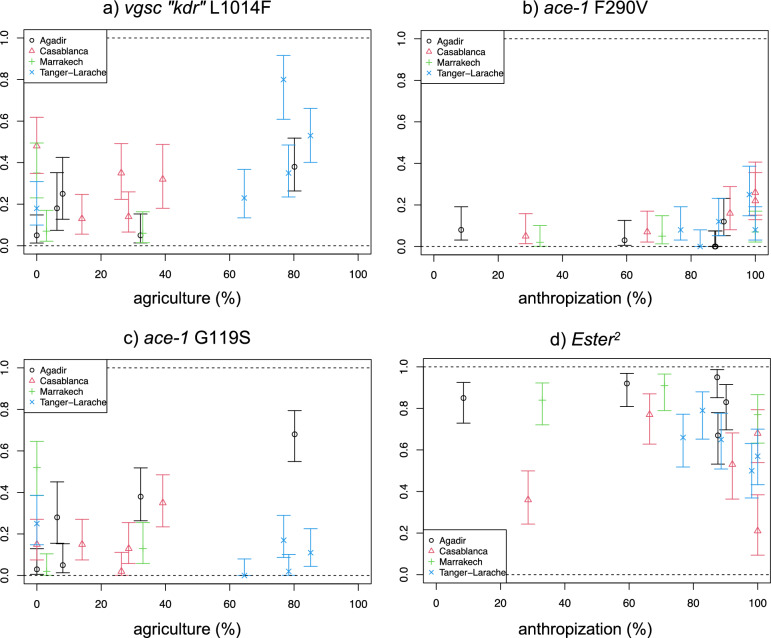
Effect of anthropization and agriculture on resistance allele frequencies in Morocco. The frequencies (with their 95% confidence intervals) of each resistance mutation (**a** *kdr*, **b** *ace-1 F290V*, **c** *ace-1 G119S* and **d** *Ester*^*2*^) are presented for the three to five populations of each region (different colors), according to either the percentage of agricultural land (**a** and **b**) or the percentage of anthropized land (**c** and **d**), depending on the main effect detected in PCA (see text, Fig. 2 and Table 2)

We then more formally tested the significance of these correlations using GLMs. We assessed how the frequencies of the resistance mutations were affected by (i) the percentage of agricultural land coverage (*%agri*) for *kdr* and *ace-1* G119S and (ii) for the two other resistance alleles, the percentage of anthropized lands (defined here as *%anthrop* = *%agri* + *%urb*, i.e. *%anthrop* = 100%-*%nat*). For all resistance mutations, the plots show variations between the transects considered (Fig. 3), which was confirmed by the finding of significant interactions between the variables *Region* (i.e. the transects) and *Land* (i.e. the percentages of agricultural or anthropized lands; likelihood ratio tests, LRT, *χ*^*2*^ = 8.6, 13.8, 30, 72, *df* = 3, *p*-values = 0.035, 0.003, < 0.001, < 0.001, for *Ester*^*2*^, *ace-1* F290V, *kdr* and *ace-1* G119S, respectively). We thus analyzed the effects of land usage independently for each transect (Table 2). These analyses confirmed the heterogeneity of the effects for the different transects and resistance mutations: (i) for *kdr* and *ace-1* G119S, the frequencies of the resistance mutations increase with the percentage of agricultural lands, and the effect is significant for all transects (although for Casablanca the *p*-value is no longer significant after Bonferroni correction; Table 2a and Fig. 3a, c); (ii) for *ace-1* F290V, the frequency of the resistance mutation increases with the percentage of anthropized lands, but the effect is significant only for the Casablanca transect (for Tanger the *p*-value is no longer significant after Bonferroni correction; Table 2b and Fig. 3b); (iii) finally, for *Ester*^*2*^, there is no significant correlation between the frequency of the resistance mutation and the percentage of anthropized lands (for Tanger the *p*-value is no longer significant after Bonferroni correction; Table 2b and Fig. 3d).

**Table 2:** Effects of agriculture and anthropization on resistance mutation frequencies for each Moroccan region

Region	(a) Agriculture effect		(b) Anthropization effect	
	*kdr* L1014F	*ace-1* G119S	*ace-1* F290V	*Ester*^*2*^
Agadir	***6.3* × *10***^***–5***^	***2.6* × *10***^***–17***^	0.24	0.39
Casablanca	*0.037*	*0.043*	***2.2* × *10***^***–4***^	0.22
Marrakech	***0.002***	***0.006***	0.17	0.33
Tanger	***1.2* × *10***^***–5***^	***0.002***	*0.015*	*0.017*

For each resistance mutation, the *p*-values for GLM likelihood ratio tests (see Material and Methods) for the effect of **a** agricultural land coverage in a 2.5-km radius around the sampling site or **b** anthropization, i.e. urban + agricultural land coverage, are indicated. For each resistance mutation, the effect tested is based on the PCA results (Fig. 1). *P*-values are italicized when < 0.05 and bolded when still significant after sequential Bonferroni correction [54]

Our study thus reveals a clear impact of human activities and/or densities on most of the insecticide resistance mutations. As in other places in the World, large modifications of the natural environment by human activities or anthropization (deforestation, urbanization, agriculture, industrial activity, etc.) have affected Morocco: the last decades witnessed a fast and extended urbanization coupled with a fast regional economic development. This led to the introduction of many residuals from human activities in the environment, from the industrial and agricultural sectors in particular. Agriculture is indeed one of the pillars of the Moroccan economy, and massive pesticide usage remains strategic to limit the risk of crop loss. However, the increased concentration of people in cities also resulted in an increased use of domestic pesticides. Finally, the more people, the more local governing bodies are prompted to use insecticides for pest and disease control. The density of humans is also reflected in our categorization, from high populated urban areas to less populated agricultural areas, and then natural areas where the density of humans is the lowest. Thus, while we cannot disentangle here the effects of direct disease control targeting mosquitoes from the effects of other human residuals selecting resistance genes, our land use categorization does represent a gradient of human pressure.

In the present study, we show a clear and positive correlation with agricultural lands in particular for the two most frequent target site mutations (*kdr* and *ace-1*) described in mosquitoes. This is less clear for the more generalist detoxifying esterase, a metabolic resistance mechanism; the constitutively overexpressed *Ester*^*2*^ allele indeed appears pervasive at high frequencies in most populations (> 0.6 in most samples), regardless of the local land usage.

These differences are probably due to the higher specificity of the target site mutations (as suggested in [41, 44]. They are probably mostly selected by pesticide molecules used for agriculture, which are of the same families as those used for vector control (PYR, OP and CX); by contrast, metabolic resistance mutations are probably more easily selected by a wider range of residuals. For *ace-1* G119S, frequencies indeed remain relatively low compared to what was observed in Southern France when OP was used for vector control (respectively mostly < 0.5, Fig. 3, vs > 0.9 [59]); contrarily, *Ester*^*2*^ frequencies are often > 0.8, which is much higher than what was observed after OP stopped being used for vector control in Southern France (< 0.4% [41]. Taken together, these suggest an ongoing but relatively mild OP insecticide pressure, as witnessed by *ace-1* G119S, but much higher selective pressures from other compounds found in the environment, acting on *Ester*^*2*^.

Several studies have reported a role of the use of pesticides for agriculture in the selection of insecticide resistance in pathogen vectors, particularly for the malaria vectors of the genus *Anopheles* [20, 35, 36, 59–64]. For example, in *An. gambiae* *s.l.*, resistance to permethrin and DDT is higher in cotton-growing areas in Burkina Faso and Benin [65], and urban agriculture appears to be the main driver of insecticide resistance in Cameroon [35]. A review of the literature confirmed that crop pest control represents a strong selective pressure, on top of vector control, promoting resistance to PYR [20].

However, other residuals from human activities (including domestic usage on insecticides) can also result in positive selection of insecticide resistance mutations. A recent study in Tunisia showed that *C. pipiens* *s.s. *populations exposed to higher levels of anthropogenic pollutants exhibit stronger selection signals for OP and CX targets (*ace-1* and *Ester* loci; [38], and another showed that *Culex quinquefasciatus* OP and CX resistance in Ivory Coast and Burkina Faso was due to the domestic use of insecticides rather than to agricultural pesticides [66]. Although their effects are quite difficult to individualize, so that the topic remains largely unexplored, the presence of pollutants other than those used for vector control in breeding habitats of mosquitoes can indeed select for resistance [42], and a few studies showed that exposition to common urban pollutants increases mosquito tolerance to insecticides [67, 68]. Our study suggests that species of the *C. pipiens* *s.l. *complex, due to their ecological preferences for breeding sites concentrating all sorts of residuals, are probably exposed to a wider range of xenobiotics than other species of mosquitoes (as found in a previous study in Mayotte [22]), which could impose a positive selection on mutations originally providing resistance to vector control. In that sense, *C. pipiens* *s.l.* species are interesting sentinels to indirectly survey the intensity of anthropogenic residual production around their breeding sites.

### No clear trends for the short-term dynamics of insecticide resistance genes in Morocco

**Fig. 4:**
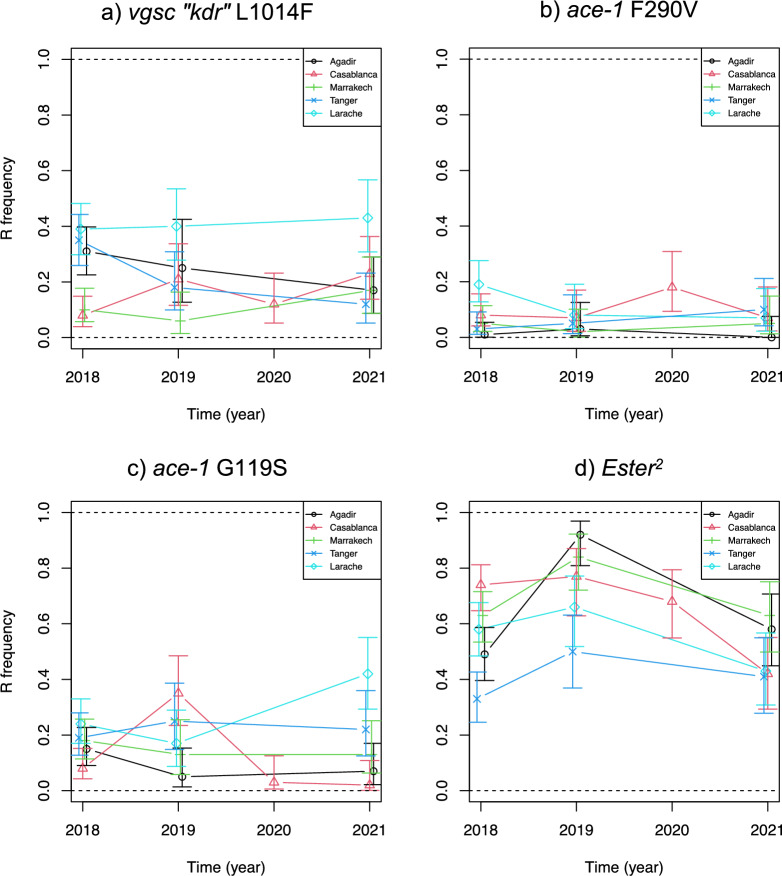
Resistance dynamics in Morocco from 2018 to 2021. The dynamics of each resistance mutation frequency (**a** *kdr*, **b** *ace-1 G119S*, **c** *ace-1 F290V* and **d** *Ester*^*2*^) are presented with their 95% confidence intervals for the focal populations of each region (same colors as Fig. 3; the sample sites are the same as in Arich et al. [45], see Table 1, bolded and italicized)

Another goal of the present study was to address the dynamics of insecticide resistance in *C. pipiens* *s.s. *in Morocco. To this end, we followed up on a first evaluation in 2018 of resistance and resistance allele frequencies in five cities [48] by resampling and analyzing the same breeding sites in 2019 and in 2021 (and in 2020 for Casablanca; Table 1 and Fig. 4).

While there are variations from one year to the other, these dynamics do not show any clear trend over the 4 years, for any of the resistance alleles, or in any region. The only noticeable patterns are observed (i) for *Ester*^*2*^, where a spike of resistance all over Morocco was observed in 2019, with no obvious explanation, and (ii) for *ace-1* G119S in Larache, where the frequency of the resistance mutation increased from 0.2 to 0.4 between 2019 and 2021. While this increase might result from increased insecticide usage, it seems unlikely as no increase is seen for *Ester*^*2*^, which still confers resistance to similar insecticides, including the larvicide temephos (OP) used for vector control in Morocco.

Some previous studies (on various mosquitoes or other species) tended to indicate that resistance gene frequencies to insecticide dose modifications can change relatively quickly (over a few years), either increasing when insecticides start to be used (e.g. [69–71]), decreasing after insecticide usage stops (e.g. [41, 43, 72, 73]) or even adjusting to more limited variations, even seasonal [74]. Overall, our data thus suggest that, during the study period, little to none significant change happened in either vector control intensity or other anthropogenic residual emissions.

However, these data also show that resistance alleles remain present all over Morocco (Figs. 3 and 4); *kdr* and *ace-1* G119S are found at relatively high frequencies (in the range of, or even lower than, what was observed in other countries (e.g. [41, 44, 59]), and *Ester*^*2*^ displays much higher frequencies, higher than what was generally observed in other countries (e.g. [41, 44). Little oversight is applied for the use of insecticides in this country, local administrations being mostly responsible for the choices in terms of quantities used [48]. Should concerted action nevertheless be taken, resistance is already under selection by multiple sources, and may further increase rapidly, putting any plan not designed carefully enough in jeopardy.

## Conclusions

Our study provides further evidence for the role of anthropic activities in the selection and maintenance of mutations responsible for resistance to insecticides in mosquitoes. This is particularly true for agricultural lands, where pesticides often affect non-targeted species [36, 39, 60, 61], but other residuals, including urban pollution, use of domestic insecticides in large cities and vector control, also appear implicated. The main worry here is that when unintended selections result in increasing resistance in a mosquito vector of human diseases, as is the case for *C. pipiens* in Morocco (e.g. WNV), the consequences can be dramatic and result in the spread of these diseases. The control of resistance is indeed a central and complex concern for all vector control programs, but the disparate origins of insecticide resistance selection pressures make it even more difficult. Managing resistance to maintain control of vectorial diseases will thus require comprehensive and integrated strategies, which should consider all the major sources of resistance selection, not only vector control. They must thus be organized among various authorities, not only from the medical fields, but also from industry and agriculture, economy, city planning, waste management and ecology. They will also require long-term monitoring of different indicators to detect new sources of selection and manage those already present, as the ever-growing anthropization of the ecosystems indeed has complex consequences. However, in this dire context, the original habitat preferences and diverse resistance mechanisms of *C. pipiens* could turn to our advantage, as they are a powerful tool to achieve comprehensive and integrated surveys of territories, i.e. this mosquito could become an ecological sentinel.

### Supplementary Information

**Additional file 1: **The data used for the analyses are given in two tables: Data 1 for the geographical survey and the selection analyses related to land usages; Data 2 for temporal analyses.

## Data Availability

All the data are provided in Additional File 1.
